# Use of cell-free DNA in early detection of lung cancer: a proof-of concept study

**DOI:** 10.1186/s12885-026-15790-0

**Published:** 2026-02-25

**Authors:** Larissa L. White, Mahesh Maiyani, LeeAnn M. Quintana, John D. Powers, Lindsay E. Nichols, Blythe Dollar, Ruth Bedoy, America Elias Martinez, Heather Spencer Feigelson

**Affiliations:** Kaiser Permanente Colorado Institute for Health Research, 16601 East Centretech Parkway, Aurora, CO 80011 USA

**Keywords:** Lung neoplasms, Early detection of cancer, Blood-based biomarker, Cell-free DNA, Lung cancer screening

## Abstract

**Background:**

Low-dose computed tomography (LDCT) remains the only validated modality for lung cancer screening, yet participation is suboptimal. Blood-based assays could eventually complement LDCT, but their performance for detecting early-stage disease in screening populations is uncertain. This study evaluated how well a cell-free DNA blood-based screening test can detect early-stage lung cancer in patients with LDCT screen-detected or incidental lung nodules.

**Methods:**

We recruited patients via email with upcoming LDCT appointments whose results were classified under the Lung-RADS categories of 1–4. Guardant Health LUNAR^®^ test collection kits were mailed to the participant. Participants provided a blood sample on the day of their LDCT visit, or within 30 days of the scan. Sensitivity and specificity were computed using the tumor registry as the gold standard and compared to assay results from the blood draw date closest to the date of diagnosis of cancer, but prior to receipt of any treatment.

**Results:**

Of the 765 participants, 18 lung cancers were diagnosed. Most of the study population had a previous lung cancer screen. Eleven cases were diagnosed with stage 1 disease (68.8%). The sensitivity was 43.8% (95% CI:19.4–68.1%) and the specificity was 84.1% (95% CI: 81.5–86.7%), which resulted in a false positive rate of 15.9%. The negative predictive value was 98.6% and the positive predictive value was 5.6%. Exploratory stage-adjusted sensitivity was 75.4% (95% CI:63.6–87.2%).

**Conclusions:**

The cfDNA assay demonstrated moderate specificity but limited sensitivity. Given the false positive rate of 15.9%, our results suggest that cfDNA-based assays for lung cancer may play a role as tools in combination with existing screening strategies pending continued validation. This proof-of-concept study is an important step in determining the feasibility of blood-based testing for early lung cancer detection.

**Supplementary Information:**

The online version contains supplementary material available at 10.1186/s12885-026-15790-0.

## Background

Lung cancer is by far the leading cause of cancer death in the US, accounting for about 1 in 5 of all cancer deaths. Each year, more people die of lung cancer than of colon, breast, and prostate cancers combined [1]. Lung cancer screening with low-dose computed tomography (LDCT) has been shown to reduce lung cancer mortality among people at high risk [2–4]. The American Cancer Society (ACS) and the US Preventive Services Task Force (USPSTF) began recommending lung cancer screening using LDCT in 2013 for individuals aged 55–80 years with a 30 pack-year smoking history who currently smoke or have quit within the past 15 years [4]. In 2021 and 2023, the USPSTF and ACS respectively expanded their eligibility criteria to include people aged 50–54 years and those with a 20–29 pack-year smoking history [5–6].

Despite the demonstrated benefits of lung cancer screening, the uptake of LDCT screening remains low. In 2020, it was estimated that only 6.5% of people eligible for screening in the US received a screening exam [7]. Patient-, provider- and system-level barriers persist in lung cancer screening, including limited awareness of screening guidelines, misconceptions about risk, stigma related to smoking history, competing clinical priorities, limited imaging facilities in rural areas, and long wait times in urban areas [4–15]. Together these barriers result in low overall coverage of screening, even when uptake among those participating in lung cancer screening programs may be higher. It has been suggested that blood-based tests could support existing cancer screening and diagnostic approaches [16, 17], especially in lung cancer screening where patients may be reluctant to undergo imaging, continue adherence to their LDCT intervals, or face such barriers to receiving guideline-recommended LDCT. We conducted a proof-of-concept prospective study to evaluate whether a cell-free DNA (cfDNA) blood-based screening test could detect early-stage lung cancer in patients with LDCT screen-detected or incidental lung nodules.

## Methods

Starting in 2014, Kaiser Permanente Colorado (KPCO) implemented a lung cancer screening program to identify eligible patients and offer them LDCT screening free of charge. This program tracks patients with lung nodules, and in particular, high-risk nodules that should be monitored or evaluated for cancer. For this study, we queried the electronic health record (EHR) weekly to identify upcoming appointments for LDCT and new radiology reports of recent LDCT scans among patients who appeared to be eligible based on data in the EHR.

We limited enrollment to patients whose LDCT results were classified under the Lung-RADS (version 1.0) [18] categories of 1–3 (recommended follow-up at 12-month intervals) or category 4 (“suspicious”; recommended follow-up at 3-month intervals). This was to ensure we had both low risk and high-risk patients included in the study who had received their guideline-recommended screening. Further, participants had to be active KPCO members 35 years of age and older and able to understand and provide consent in English. We excluded patients with lung nodules *≥* 30 mm in which malignancy likelihood is already high, those with a history of any hematologic malignancy, history of invasive malignancy within the past 5 years, previous biopsy of the nodule of interest, currently pregnant, or known dementia or cognitive impairment with inability to provide informed consent without a legally authorized representative.

Because most of the study enrollment period took place during the initial years of the COVID-19 pandemic, it was important to minimize in-person interactions; thus, recruitment was primarily among patients with an email address listed in the EHR (approximately 80% of the adult patient population). KPCO patients with upcoming LDCT appointments were invited to participate in the study via email. Follow-up phone outreach was incorporated later in the study to improve the response rate. A link to the online informed consent form and a baseline survey were provided. Patients could request written materials by mail and could also consent via phone.

After informed consent was attained, Guardant Health LUNAR^®^ test collection kits were mailed to the participant with sample collection instructions provided. Participants were asked to provide a blood sample on the day of their LDCT visit, or within 30 days of the scan. Clinic staff collected the samples using Streck cfDNA tubes and mailed the kits back to Guardant Health using an overnight courier. A similar process for sample collection was used for any follow-up LDCT appointment occurring between enrollment and September 30, 2022, and any biopsy or diagnosis of cancer between enrollment and September 30, 2022. Samples were only collected at LDCT scans or lung biopsies ordered by the participant’s physician as part of routine clinical care and based on USPSTF guidelines [19]. Thus, participants deemed low risk by clinical guidelines may only have a single blood sample collected at baseline, while participants with lung nodules classified as “suspicious” may have LDCT every three months, and therefore, blood samples would be collected at multiple timepoints during the study.

Assay results were not returned to participants or providers because the test was investigational and not validated for clinical decision‑making. All participants continued guideline‑recommended LDCT screening and diagnostic follow‑up per their clinicians. LDCT surveillance remained the sole basis for clinical follow‑up, ensuring no participant was deprived of standard‑of‑care imaging.

### Data collection

The baseline survey was created for this study and collected data on smoking history, personal and family history of cancer, demographics, passive tobacco exposure, and occupational history (Supplementary Material 1). Data on LDCT and pathology results were pulled from the EHR. Additionally, KPCO maintains a centralized tumor registry of all cancer patients diagnosed since 1987 and who are members in the Denver/Boulder service area. New cases are identified monthly using specific cancer-related International Classification of Diseases (ICD) 10 codes from three sources; pathology, external claims and internal appointments which are then imported into the registry database using an automated case-find module. The tumor registry was used to confirm all reported cancers, to collect histopathologic data, and was queried at the end of the study to ensure no cancer diagnoses were missed during the study. Chart review was performed by certified tumor registrars to confirm diagnosis if needed.

### Sample processing

Whole blood samples (30 to 80 ml) were collected in Streck cfDNA tubes and shipped at ambient temperatures overnight to the central laboratory for analysis (Guardant Health). The required volume reflects the test’s analytic components described below, which necessitate larger plasma yields. While higher than standard venous draws, this volume is consistent with other cfDNA‑based research protocols. All samples were received in the central laboratory blinded to clinical findings. Samples were processed to plasma and stored at -80 degrees Celsius until analysis.

The test under assessment is a cfDNA blood-based assay for the detection of lung cancer. The panel interrogates cfDNA genomic alterations, aberrant methylation status, and fragmentomic patterns. Results are integrated into a binary “abnormal signal detected” (positive test) or “normal signal detected” (negative test). Binary results were reported to the KPCO research team, where they were associated with the clinical outcomes for analysis. Results were not returned to participants or entered into the EHR as this was a proof-of-concept study, and participants were adhering to their recommended lung cancer screening.

### Statistical analysis

All analyses included only participants who developed lung cancer and those who remained cancer free; people who developed other cancers were excluded from these analyses given that the purpose of this study was to detect early-stage lung cancer in participants with LDCT screen-detected or incidental lung nodules. We excluded two samples collected more than 30 days after the LDCT (66 and 86 days, respectively). The rationale for the 30-day cut-off point was to maintain proximity between the LDCT and the assay blood draw. Hypothetically, if the time range between the LDCT and assay blood draw were further apart, the assay result could potentially reflect a physiological finding that was not present at the time of LDCT. We used Chi-square and Wilcoxon rank-sum tests to compare differences between those who developed lung cancer and those who did not. Sensitivity and specificity were computed using the tumor registry as the gold standard and compared to assay results from the blood draw date closest to the date of diagnosis of cancer, but prior to receipt of any treatment. For participants who remained cancer-free, the assay results used for comparison was the sample collected closest to the end of follow-up, which could be the single sample collected at baseline.

Since our study population had a high rate of prior LDCT screening and thus a stage distribution skewed toward stage I disease, we conducted an exploratory stage‑adjusted sensitivity analysis using stage distributions from the SEER database (2012–2021) [20]. This method re‑weights observed sensitivity according to SEER stage frequencies; however, it does not change the number of cancers actually detected or missed in this cohort. Sensitivity estimates depend heavily on where in the diagnostic pathway cancers are detected; sensitivity among clinically diagnosed cases or enriched stage I cohorts can misrepresent true preclinical or population-level sensitivity [21–23]. Depending on the stage distribution and timing of detection, study results can produce potentially biased sensitivity metrics [21–23]. With this in mind, providing a stage‑weighted estimate (using external stage distributions) can contextualize the observed sensitivity. This adjustment is intended only as a contextual comparator, not as a substitute for empirically observed sensitivity in an unscreened population [21–23].

## Results

**Fig. 1:**
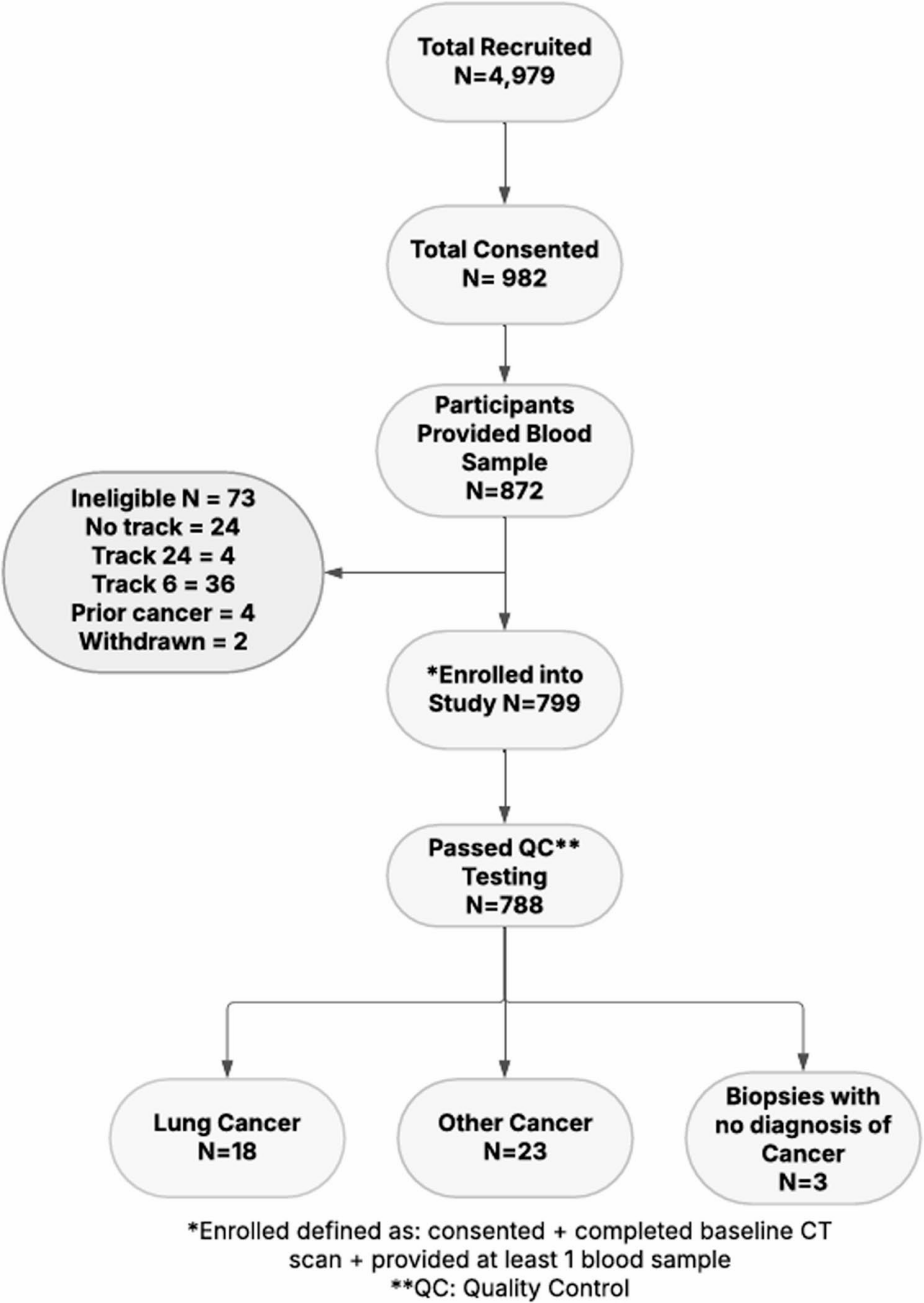
Study enrollment and outcomes

Enrollment for this study began 9/11/2020 and ended 8/31/2022. All samples were collected by 12/31/2022. Diagnosis of cancer was tracked until 12/31/2022. As shown in Fig. 1, 799 people enrolled in the study (defined as providing informed consent, completing a baseline LDCT scan, and providing at least 1 blood sample), of whom 788 also passed all quality control tests for the assay. Eighteen lung cancers were diagnosed, 13 (72%) of which were stage 1. Table 1 displays the characteristics of the study population. Those diagnosed with lung cancer were older (mean age = 71.8) than those who were cancer free (mean age = 67.9, *p* = 0.023) and had more pack-years of tobacco use (57.4 vs. 43.2, *p* = 0.076). The frequency of a prior cancer was statistically significantly higher among those diagnosed with lung cancer (44.4% vs. 10.2%, *p* < 0.0001). Most of the study population had a previous lung cancer screen (78.6% of those cancer-free and 55.6% of cases, *p* = 0.02). Over half of the population was up to date on colon cancer and mammography screening guidelines. Participants were mailed collection kits and asked to have their blood drawn within 30 days of their scan, preferably on the day of the scan. Of the 18 lung cancer cases, 5 (27.8%) had samples collected on the day of their LDCT, 10 (55.6%) were collected +/-14 days, and 16 (88.9%) were collected within 30 days (data not shown).

**Table 1:** Characteristics of study population (*N* = 765)

	**Cancer Free (N=747)**		**Lung Cancer (N=18)**		
**Characteristic**	*N*	%	*N*	%	***P*** **-value** ^a^
Age, years (mean, SD)	67.9	(7.5)	71.8	(6.3)	0.023
Cigarette Pack Years (mean, SD)	43.2	(25.4)	57.4	(35.6)	0.076
Charlson comorbidity index	1.5	(1.6)	2.1	(1.7)	0.080
Sex					0.946
Female	421	56.4	10	55.6	
Male	326	43.6	8	44.4	
Self-reported Race/Ethnicity					0.744
Non-Hispanic Black	20	2.7	0	0	
Hispanic	41	5.5	1	5.6	
Non-Hispanic White	658	88.1	17	94.4	
Other	28	3.7	0	0	
Personal history of cancers					
No	656	87.8	9	50.0	< 0.0001
Yes	76	10.2	8	44.4	
Unknown	15	2.0	1	5.6	
Smoking status					0.867
Current	289	38.7	8	44.4	
Former	402	53.8	9	50.0	
Never	56	7.5	1	5.6	
Lung cancer screening prior to consent					0.020
No	160	21.4	8	44.4	
Yes	587	78.6	10	55.6	
Colon cancer screening prior to consent					0.973
No	335	44.8	8	44.4	
Yes	412	55.2	10	55.6	
Mammography prior to consent (women, *N* = 431)					0.0471
No	124	29.5	4	40.0	
Yes	297	70.5	6	60.0	

^a^Wilcoxon rank-sum for means; Chi-square tests for categories

Table 2 shows the stage distribution stratified by prior lung screening with LDCT. Eleven cases were diagnosed with SEER summary stage 1 disease (68.8%), 7 of whom had at least one prior LDCT exam. Of those who had no prior LDCT screening, 42.8% had stage > 1, compared to 22.2% of those with a prior screen. Of the 11 cases with SEER summary stage 1, seven were adenocarcinoma, two were non-small cell carcinoma (unspecified), and two were squamous cell carcinoma. Six of the 11 SEER summary stage 1 cases had tumors > 1 – ≤ 2 cm. Of the two cases with SEER summary stage 2, one was adenocarcinoma and the other was small cell carcinoma. Of the three cases with SEER summary stage 3 or 7, all cases were small cell carcinoma (data not shown). Table 3 shows the diagnostic yield of the LUNAR^®^ assay in the study population, where 16.5% of participants had a positive test result.

**Table 2:** Stage distribution for lung cancers diagnosed within 30 days of specimen collection by history of lung cancer screening (*N* = 16)

	Prior Lung Cancer Screening		
SEER summary stage	No *N* (%)	Yes *N* (%)	Total *N* (%)
1	4 (57.1%)	7 (77.8%)	11 (68.8%)
2	1 (14.3%)	1 (11.1%)	2 (12.5%)
3	1 (14.3%)	1 (11.1%)	2 (12.5%)
7	1 (14.3%)	0	1 (6.2%)
Total	7	9	16

**Table 3:** Diagnostic yield of lung cancer confirmed by the tumor registry in the screened study population (*N* = 763)

	Lung Cancer (Tumor Registry)			
Blood-Based Test	Yes *N* (%)	No *N* (%)		Total
Positive	7 (5.6%)		119 (94.4%)	126
Negative	9 (1.4%)		628 (98.6%)	637
Total	16 (2.1%)		747 (97.9%)	763

Table 4 shows the sensitivity, specificity, positive predictive value (PPV) and negative predictive value (NPV) of the LUNAR^®^ assay using the tumor registry as the gold standard. The sensitivity for lung cancer was 43.8% (95% CI:19.4–68.1%) and the specificity was 84.1% (95% CI: 81.5–86.7%), which resulted in a false positive rate of 15.9%. The NPV was 98.6%, while the PPV was 5.6%. Because our study population is insured, has access to screening, and most had received a prior LDCT, we used SEER stage data for lung cancer between 2012 and 2021 to stage-adjust our observed sensitivity as described in the Methods section. The stage-adjusted sensitivity was 75.4% (95% CI:63.6–87.2%) (Table 4). This is similar to the sensitivity if the calculation is limited to stage > 1 cases (sensitivity = 80.0%); however, the restricted analysis which is limited to stage > 1 cases as opposed to stage-adjusted sensitivity is based on only five cases.

**Table 4:** Sensitivity, stage-adjusted sensitivity, specificity, positive and negative predictive values of the blood-based test for lung cancer diagnosed within 30 days of blood sample collection (*N* = 16)

Statistic	Estimate	95% Confidence Limits	
Sensitivity	0.438	0.194	0.681
Stage-Adjusted Sensitivity^a^	0.754	0.636	0.872
Specificity	0.841	0.815	0.867
Positive Predictive Value	0.056	0.016	0.096
Negative Predictive Value	0.986	0.977	0.995

^a^Stage-adjusted sensitivity utilized SEER stage data for lung cancer 2012–2021

## Discussion

We conducted a proof-of-concept prospective study to assess the feasibility of using a blood-based cfDNA test to detect lung cancer. Over the 24 months of the study, 18 cases of lung cancer were detected by LDCT and confirmed by the tumor registry (approximately 2% incidence). Most of the cases (68.8%) in this study were diagnosed at stage I. The cfDNA assay achieved a sensitivity of 43.8% (95% CI:19.4–68.1%) and specificity of 84.1% (95% CI: 81.5–86.7%). While these results are consistent with or modestly higher than the performance of other blood-based tests and predictive models, the assay missed more than half of lung cancers [17, 24–25]. The false positive rate was 15.9%, which is within the lower range reported for baseline LDCT (7.9–49.3%) [26]. The PPV was 5.6% and the NPV was 98.6%, which likely reflects the study sample of a highly screened, insured population, most of whom had undergone prior LDCT screening with a relatively low prevalence of lung cancer. Since NPV is driven by disease prevalence, the high NPV of 98.6% should not be interpreted as evidence that the test can reliably exclude cancer in an unscreened or higher-risk population. For comparison, LDCT has a reported sensitivity from 59% to 100%, specificity from 26.4% to 99.7%, PPV from 3.3% to 43.5%, and NPV from 97.7% to 100% [5, 27–29]. We used SEER-derived stage distributions to adjust for stage which resulted in improved sensitivity of 75.4%. However, this approach has important limitations and should be interpreted with caution. The adjusted estimate reflects the performance we might expect in a population with a stage distribution similar to the national average, but it does not change the number of cancers actually missed in this study (9 out of 16 cases). Therefore, the stage‑adjusted sensitivity should be considered exploratory and hypothesis‑generating rather than definitive evidence of improved assay performance.

While the literature on clinically validated blood-based methods for lung cancer detection and triage is low, there are primarily three purposes for this approach: (1) early detection and diagnosis; (2) risk prediction and stratification; and (3) differentiation between benign and malignant pulmonary nodules. This study’s cfDNA findings were similar to those reported for other lung cancer-based biomarkers, such as autoantibodies [30–31]. A study of 447 participants utilized autoantibody testing to determine the malignancy status of pulmonary nodules in two prospective cohorts [32]. The study reported sensitivity of 16%, specificity of 90%, PPV of 66%, and a false positive rate of 10%. The authors concluded that autoantibody testing could potentially streamline diagnostic workup for high-risk patients. Additional research has highlighted the advantages and pitfalls of biomarkers for lung cancer detection – considering analytes like cfDNA as a tool for overcoming barriers to LDCT screening rather than replacement of LDCT screening [33–34]. There have been significant strides in biomarkers for early detection of other screenable cancers, specifically colorectal cancer. In a cohort study assessing the performance of a blood-based cfDNA test in 7,861 participants eligible for colorectal cancer screening, the assay achieved 83% sensitivity for colorectal cancer, 90% specificity for advanced neoplasia, and 13% sensitivity for advanced precancerous lesions [16]. As technology continues to improve, there is reasonable expectation that blood-based test performance for lung cancer detection may be able to achieve the same success.

This proof-of-concept study is an important early step in determining the feasibility of blood-based testing for early lung cancer detection and risk stratification. We demonstrated that a cfDNA assay may be a feasible addition to the lung cancer screening and diagnostic paradigm. However, several limitations should be noted. Our population of insured, screen-compliant people resulted in a downward shift in stage at diagnosis that is not representative of the US population. Future studies should prioritize enrolling people at the time of their first LDCT. More than half (9/16) of the lung cancer cases in this study had been screened before; conducting a similar study in an unscreened population would provide a better assessment of the accuracy of a blood-based test and its feasibility in conjunction with LDCT and established diagnostic workflows. This study utilized e-mail outreach as its primary form of recruitment. About 20% of our membership do not have an e-mail address, which may have imparted selection bias into our final cohort. Ideally, blood samples would be collected on the same day as the LDCT scans, but limitations to our study design because of COVID-19 precautions often led to gaps between the scan and specimen collection.

## Conclusions

This prospective study assessed the feasibility of a blood-based cfDNA assay to detect lung cancer. The test resulted in a sensitivity of 43.8% and specificity of 84.1%, detecting fewer than half of the lung cancers identified during the study period. Although an exploratory stage‑adjusted analysis yielded a higher estimated sensitivity of 75.4%, this model‑based approach does not change the number of cancers missed and should be interpreted cautiously. Given the false positive rate of 15.9%, our results suggest that cfDNA-based assays for lung cancer may play a role as tools in combination with existing screening strategies pending continued technological validation and advancement. Further study is warranted to assess the assay’s clinical accuracy and utility in unscreened populations, particularly populations who face barriers to guideline-recommend LDCT.

## Supplementary Information

Supplementary Material 1.

## Data Availability

The datasets generated and/or analyzed during the current study are not publicly available due to the privacy of individuals and health system members that participated in the study, but derived data are available from the corresponding author on reasonable request.

## Abbreviations

LDCT: low dose computed tomography
ACS: American Cancer Society
USPSTF: United Stated Preventive Services Task Force
cfDNA: cell free deoxyribonucleic acid
KPCO: Kaiser Permanente Colorado
EHR: electronic health record
Lung RADS: Lung Screening Reporting & Data System
ICD: International Classification of Diseases
SEER: Surveillance, Epidemiology, and End Results Program
PPV: positive predictive value
NPV: negative predictive value
