# Supplementary material for: Use of cell-free DNA in early detection of lung cancer: a proof-of concept study
Source: BMC Cancer. 2026 Feb 25;26:432. doi: 10.1186/s12885-026-15790-0 (PMC13040859; doi:10.1186/s12885-026-15790-0)
Supplement: Supplementary file 1 — Supplementary Material 1. [file 12885_2026_15790_MOESM1_ESM.pdf]

Baseline Survey

Please complete the survey below, and answer to the best of your ability. If you feel more comfortable not answering a question, please choose "prefer not to answer".

If you have any questions while completing this survey, please call the Lung Screening Study team at 303-636-3242 or email lung-screening-study@kp.org

Thank you!

Thank you for taking the time to fill out this brief survey. Your responses will give us important information about you, your exposure to tobacco, and your family history.

1. What best describes your race or ethnicity? (mark all that apply)
   - ☐ Hispanic
   - ☐ Black or African American
   - ☐ Asian
   - ☐ Native American
   - ☐ White
   - ☐ Other (please specify)
   - ☐ Prefer not to answer

   ____

2. Have you ever been diagnosed with cancer (please do not include non-melanoma skin cancer)?
   - ◯ Yes
   - ◯ No

   If yes, what type of cancer? ______________________

   Year of diagnosis: ______________________

3. What is the nature of the business or industry where you have worked during the majority of your life? (Please select one)
   - ◯ Active duty military
   - ◯ Architecture and engineering
   - ◯ Arts, design, entertainment, sports and media
   - ◯ Building and grounds cleaning and maintenance
   - ◯ Business and financial operations
   - ◯ Community and social services
   - ◯ Computer and mathematical sciences
   - ◯ Construction
   - ◯ Education, training, and library
   - ◯ Farming, fishing, and forestry
   - ◯ Food preparation and serving
   - ◯ Healthcare
   - ◯ Homemaker
   - ◯ Industrial Production
   - ◯ Installation, maintenance, and repair
   - ◯ Legal services
   - ◯ Life, physical, and social sciences
   - ◯ Management
   - ◯ Office and administrative support
   - ◯ Personal care and service
   - ◯ Police, fire, and other protective services
   - ◯ Transportation and material moving
   - ◯ Sales and related services
   - ◯ Other

Please specify

____

4. Have any of your biologic family members (related to you by blood) ever been diagnosed with lung cancer?

◯ No
◯ Yes
◯ Prefer not to answer
◯ Don't know

If yes, who?

____

Smoking History

5. Have you smoked at least 100 cigarettes in your entire life?
   - ◯ No
   - ◯ Yes
   - ◯ Prefer not to answer

6. On average, how many cigarettes per day do you usually smoke (or did you usually smoke when you did smoke)?

   Note: 1 pack = 20 cigarettes

   ____

7. How old were you when you first smoked a cigarette?

   ____

8. Do you now smoke cigarettes every day, some days, or not at all?
   - ◯ Every day
   - ◯ Some days
   - ◯ Not at all
   - ◯ Prefer not to answer

8a. IF NOT AT ALL, how old were you when you last smoked cigarettes regularly?

   ____

9. How soon after you wake up do you smoke your first cigarette?
   - ◯ Within 5 minutes
   - ◯ 6 to 30 minutes
   - ◯ 31 to 60 minutes
   - ◯ After 60 minutes
   - ◯ Prefer not to answer

10. In the past year, how many times have you quit smoking for 24 hours or more? (do not count hospitalization or incarceration)

   ____

11. Have you ever smoked other type of tobacco (e.g. pipe, cigars, hookah [water pipe])?
   - ◯ No, I have never smoked these types of tobacco
   - ◯ Yes I currently smoke these types of tobacco
   - ◯ Prefer not to answer

12. Have you ever used smokeless tobacco (e.g. snuff, chew, dip, snus, betel quid)?
   - ◯ No, I have never used these types of smokeless tobacco
   - ◯ Yes, I currently smoke these type of smokeless tobacco
   - ◯ Prefer not to answer

13. Have you ever used electronic cigarettes or other forms of Electronic Nicotine Delivery Systems (ENDS) such as E-Hookah or Vape Pen?
   - ◯ No
   - ◯ Yes, more than a year ago but not in the past year
   - ◯ Yes, in the past year but more than a month ago
   - ◯ Yes, in the past month
   - ◯ Prefer not to answer

13a. If yes, in the past 30 days, on how many days did you use an E-cigarette/E-Hookah/Vape pen?

   ____

14. Have you ever smoked marijuana?
   - ◯ No
   - ◯ Yes
   - ◯ Prefer not to answer

15. About how many times in your lifetime have you smoked marijuana?

- ◯ 1 or 2 times
- ◯ 3 to 10 times
- ◯ 11 to 99 times
- ◯ 100 to 499 times
- ◯ 500 or more times
- ◯ Prefer not to answer

16. During the last 30 days, on how many days did you smoke marijuana? (If you did not use in the last 30 days, enter "00")

____

Passive Smoking Exposure

How often (measured in days per week) were you around the tobacco smoke of other people during the following various times in your life?

	N/A	< 1 day/week	1-2 days/week	3-6 days/week	Every day
Past 6 months	○	○	○	○	○
Age 60 and older	○	○	○	○	○
Age 30-59	○	○	○	○	○
Age 20-29	○	○	○	○	○
As a teenager (age 13-19)	○	○	○	○	○
As a child (age 12 and younger)	○	○	○	○	○

Passive Smoking Exposure

On days you were around others' smoke, how many hours per day were you around it during the following various times in your life?

	N/A	< 1 hour	1-2 hours	3-4 hours	5-9 hours	10+ hours
Past 6 months	○	○	○	○	○	○
Age 60 and older	○	○	○	○	○	○
Age 30-59	○	○	○	○	○	○
Age 20-29	○	○	○	○	○	○
As a teenager (Age 13-19)	○	○	○	○	○	○
As a child (Age 12 and younger)	○	○	○	○	○	○
